# Cytomegalovirus Acquisition and Inflammation in Human Immunodeficiency Virus–Exposed Uninfected Zimbabwean Infants

**DOI:** 10.1093/infdis/jiw630

**Published:** 2016-12-23

**Authors:** Ceri Evans, Bernard Chasekwa, Sandra Rukobo, Margaret Govha, Kuda Mutasa, Robert Ntozini, Jean H. Humphrey, Andrew J. Prendergast

**Affiliations:** 1 Zvitambo Institute for Maternal and Child Health Research, Harare, Zimbabwe;; 2 Blizard Institute, Queen Mary University of London, London, United Kingdom;; 3 Department of International Health, Johns Hopkins Bloomberg School of Public Health, Baltimore, Maryland

**Keywords:** HIV-exposed uninfected infants, inflammation, immune activation, cytomegalovirus, Africa.

## Abstract

Cytomegalovirus (CMV) acquisition and inflammation were evaluated in 231 human immunodeficiency virus (HIV)–exposed uninfected (HEU) and 100 HIV-unexposed Zimbabwean infants aged 6 weeks. The HEU and HIV-unexposed infants had a similarly high prevalence of CMV (81.4% vs 74.0%, respectively; *P* = .14), but HEU infants had higher CMV loads (*P* = .005) and >2-fold higher C-reactive protein (CRP) concentrations (*P* < .0001). The CMV-positive HEU infants had higher CRP than the CMV-negative HEU infants; this association disappeared after adjusting for maternal HIV load. Overall, CMV acquisition is high in early life, but HEU infants have higher CMV loads and a proinflammatory milieu, which may be driven partly by maternal HIV viremia.

Despite avoiding perinatal infection, human immunodeficiency virus (HIV)–exposed uninfected (HEU) infants have higher morbidity, mortality, and growth failure than HIV-unexposed infants [1]. The mechanisms leading to poor health outcomes are unclear, but studies have reported immune activation and a proinflammatory state in HEU infants [1]. The causes of these immunological abnormalities remain uncertain but may be related to maternal viremia and immunosuppression or increased exposure to other infections [2, 3].

Cytomegalovirus (CMV) has immunomodulatory properties, and CMV reactivation contributes to immune activation and inflammation in HIV/CMV coinfected adults [4]. Cytomegalovirus is therefore a plausible driver of immune activation in HEU infants [2, 5]. In sub–Saharan Africa, where HIV exposure remains high and CMV acquisition is almost universal in infancy, an understanding of the interactions between HIV exposure, CMV acquisition, and inflammation in early life may help to explain the poor health outcomes of HEU infants. Using a historical Zimbabwean birth cohort from the pre-antiretroviral era, we investigated the associations between maternal HIV, CMV acquisition in early infancy, and inflammation.

## METHODS

Fourteen thousand one hundred ten mother–infant pairs were recruited within 96 hours of birth to a trial of vitamin A supplementation (ZVITAMBO) in Zimbabwe between 1997 and 2001, as described previously [6, 7]. Exclusion criteria included very low birthweight (<1500g), plans to leave Harare after delivery, multiple pregnancy, or life-threatening mother/infant conditions. Infants were followed at 6 weeks and 3 months and then every 3 months thereafter. At each visit, mothers were asked to recall specific infant illness episodes in the prior 7 days. Mothers were tested for HIV using enzyme-linked immunosorbent assay (ELISA) and Western blot [6]. Those with negative tests were retested at each visit to detect seroconversion. Laboratory specimens from HIV-exposed children were stored at −70°C. The latest available laboratory specimen was tested for HIV by ELISA if the child was aged ≥18 months or DNA polymerase chain reaction (PCR) if the child was aged <18 months. Antiretroviral therapy was unavailable in public sector healthcare in Zimbabwe at the time of the trial.

### Selection of Infants

The HEU infants were defined in this substudy as infants born to HIV-positive mothers who remained HIV-negative at 6 months of age. The HIV-unexposed infants were born to HIV-negative mothers. Infants selected for this substudy met the following additional criteria: infant and maternal survival through 6 months, available infant anthropometry data, availability of 100 μL of cryopreserved plasma from the 6-week visit. All HEU infants meeting selection criteria were included. A random selection of 100 HIV-unexposed infants meeting selection criteria were included to provide comparative CMV and biomarker data.

### Detection and Quantification of Cytomegalovirus and C-Reactive Protein

Viral nucleic acid was extracted from 100 μL of plasma using the QIAamp DSP Virus Spin Kit. Each extraction included positive and negative controls. Cytomegalovirus was detected by real-time PCR on the Abbott m2000rt platform using the Abbott RealTime CMV Amplification Reagent Kit, modified for the nucleic acid extraction method we used, which therefore provided a limit of quantification of 45 copies/mL. Cytomegalovirus-positive results with viral loads below the limit of quantification were arbitrarily assigned values of 40 copies/mL. Each amplification run included positive and negative controls. C-reactive protein (CRP) was measured in plasma in singlicate by ELISA.

### Statistical Analyses

Fisher’s exact tests were used to compare categorical variables. Unpaired *t*- ests/Mann–Whitney tests (depending on distribution of data) were used to compare continuous variables. Univariable and multivariable linear regression models were used to determine the associations between CMV acquisition, CRP, and markers of maternal HIV severity. Analyses were undertaken using Prism version 6.0 and STATA version 11.1.

### Ethical Approvals

Mothers provided written informed consent to take part in the trial. The original trial and the current substudy were approved by the Medical Research Council of Zimbabwe, Johns Hopkins Bloomberg School of Public Health Committee on Human Research, and Montreal General Hospital Ethics Committee.

## RESULTS

Two hundred thirty-one HEU infants and 100 HIV-unexposed infants meeting inclusion criteria were included. The HEU and HIV-unexposed infants in this substudy were similar at baseline, but the HEU infants were born to mothers with lower mid-upper arm circumferences and hemoglobin and were more likely to be mixed fed (Table 1).

**Table 1.** Baseline Characteristics

Baseline characteristics	HIV-exposed uninfected infants (n = 231)	HIV-unexposed infants (n = 100)	*P* value
Infant characteristics			
Female sex, % (no.)	41.1 (95)	46.0 (46)	.47
Birth weight, g	2983 (493)	3034 (510)	.39
Birth length, cm	48.7 (2.5)	49.0 (2.5)	.41
Gestational age, wk, median (IQR)	39.5 (38.3–40.4)	39.7 (38.3–40.4)	.97
<37 weeks gestational age, % (no.)	9.6 (22) [230]	6.0 (6)	.39
Breastfeeding status at 3 mo, % (no.)^a^			
Exclusive	6.5 (16)	9.0 (9)	.50
Predominant	19.4 (48)	24.0 (24)	.56
Mixed	72.6 (180)	64.0 (64)	.01*
Maternal characteristics			
Age, y, median (IQR)	24.0 (21.0–28.0) [230]	23.0 (20.0–28.0)	.20
Parity, median (IQR)	2 (1–3)	2 (1–3)	.16
Vaginal delivery, % (no.)	90.9 (209) [230]	92.0 (92)	.83
Maternal midupper arm circumference,cm	25.5 (2.5)	26.2 (3.1)	.03*
Maternal height, cm	159.0 (9.2) [215]	160.0 (6.0) [92]	.35
CD4 count, cells/uL, median (IQR)	423 (298–597) [202]	…	NA
Plasma HIV RNA, copies/mL, median (IQR)	8083 (2965–26707) [184]	…	NA
Hemoglobin, g/L	113 (17) [120]	121 (19) [74]	.003*
Marital status, % (no.)			
Married/cohabiting/stable union	93.0 (214) [230]	96.0 (96)	.45
Widowed/divorced/separated	3.9 (9) [230]	0 (0)	.06
Single–never married	3.0 (7) [230]	4.0 (4)	.74
Education <8 y, % (no.)	22.1 (51) [230]	19.0 (19)	.56
Family income, USD per month, median (IQR)	73.70 (44.28–141.88)	68.77 (44.86–120.00)	.68

All results are mean (standard deviation) unless otherwise stated. Square brackets indicate number of participants with available data. Fisher’s exact tests were used to compare categorical variables. Unpaired *t* tests/Mann–Whitney tests (depending on distribution of data) were used to compare continuous variables.

Abbreviations: HIV, human immunodeficiency virus; IQR, interquartile range; NA, not applicable; USD, United States dollars; [square brackets] indicate number of participants with available data.

^a^*Statistically significant at *P* < .05. Exclusive breastfeeding: infant consumed only breast milk and prescribed medicines; predominant breastfeeding: milk main source of nourishment, but nonmilk liquids also consumed; mixed feeding: infant consumed breast milk and either nonhuman milks, such as infant formula or cows’ milk and/or food [8].

### Cytomegalovirus DNAemia

**Figure 1.**
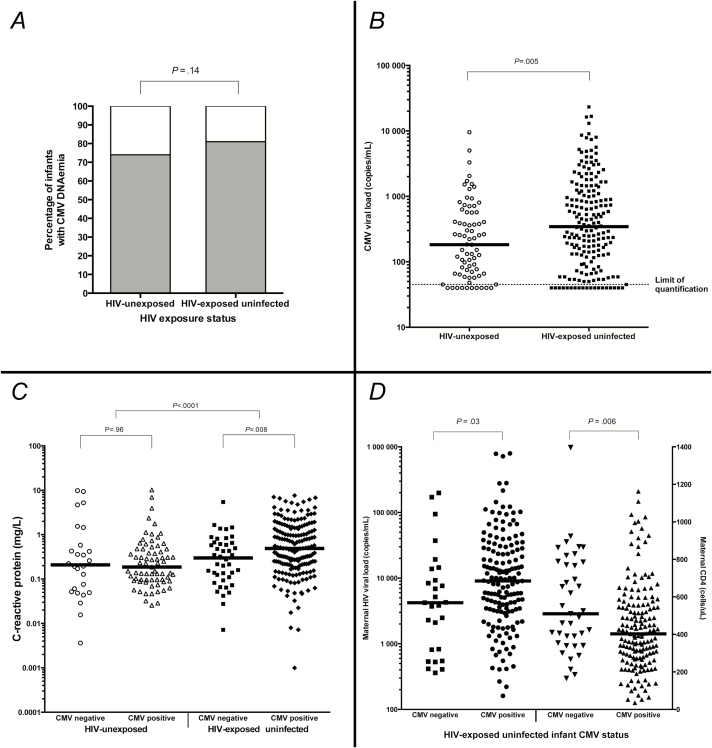
Relationship between cytomegalovirus (CMV), C-reactive protein (CRP), and human immunodeficiency virus (HIV) exposure status. *A*, Prevalence of CMV DNAemia at 6 weeks of age in HIV-unexposed and HIV-exposed uninfected infants. Proportions in each group compared using Fisher’s exact test. *B*, CMV loads in HIV-unexposed and HIV-exposed uninfected infants. Horizontal line at median. Median CMV viral loads compared between HIV-exposed and HIV-unexposed infant groups using Mann–Whitney test. Limit of quantification of CMV load: 45 copies/mL; CMV-positive samples with viral loads below the limit of quantification were arbitrarily assigned CMV load values of 40 copies/mL. *C*, C-reactive protein in infants with and without CMV acquisition by 6 weeks of age, stratified by HIV exposure category. White circles: CMV-negative HIV-unexposed infants; white triangles: CMV-positive HIV-unexposed infants; black squares: CMV-negative HIV-exposed uninfected infants; black diamonds: CMV-positive HIV-exposed uninfected infants. Horizontal line at median. All comparisons undertaken using Mann–Whitney tests. *D*, Maternal HIV disease severity in HEU infants with and without CMV acquisition by 6 weeks of age. Left *y*-axis: maternal HIV load; right *y*-axis: maternal CD4 count. Black squares: maternal viral load in CMV-negative infants; black circles: maternal viral load in CMV-positive infants; black downwards-pointing triangles: maternal CD4 count in CMV-negative infants; black upwards-pointing triangles: maternal CD4 count in CMV-positive infants. HIV-exposed uninfected infants only. Horizontal line at median. All comparisons undertaken using Mann–Whitney tests.

At 6 weeks of age, 188 of 231 (81.4%) HEU infants had CMV DNAemia compared with 74 of 100 (74.0%) HIV-unexposed infants (*P* = .14) (Figure 1A). Among infants with detectable CMV DNAemia, median CMV load at 6 weeks of age was 344 copies/mL (interquartile range [IQR] = 120–1215; range = <45–23<thin space>297) in HEU infants compared with 183 copies/mL (IQR = 66–572; range = <45–9548) in HIV-unexposed infants (*P* = .005) (Figure 1B). A higher proportion of HEU compared with HIV-unexposed infants had CMV loads >1000 copies/mL (n = 53/188 [28.2%] vs n = 9/74 [12.2%]; *P* = .006).

### Inflammation

C-reactive protein was measured in 223 of 231 (97%) HEU infants and 97 of 100 (97%) HIV-unexposed infants at 6 weeks of age. Median CRP was 0.43 mg/L (IQR = 0.17–1.23) in HEU infants compared with 0.20 mg/L (IQR = 0.09–0.48) in HIV-unexposed infants (*P* < .0001). To determine whether the higher CRP in HEU infants was related to a greater frequency of recent morbidity, we compared maternal 7-day infant illness recall between groups. The HEU infants compared with the HIV-unexposed infants had a similar frequency of diarrhea (4.7% vs 2.0%; *P* = .40), cough (37.2% vs 30.0%; *P* = .21), fast breathing (15.3% vs 16.7%; *P* = .86), difficult breathing (4.8% vs 10.0%; *P* = .14), ear discharge (0.0% vs 1.0%; *P* = .13), fever (13.5% vs 10.0%; *P* = .38), and rash (32.9% vs 36.0%; *P* = .58) but a higher frequency of oral thrush (7.8% vs 2.0%; *P* = .04) in the prior week.

Among HIV-unexposed infants, median CRP was similar between CMV-positive and CMV-negative infants (0.19 mg/L [IQR = 0.10–0.48] vs 0.21 mg/L [IQR = 0.05–0.80], respectively; *P* = .96), whereas among HEU infants, CRP was 61% higher in CMV-positive compared with CMV-negative infants (median = 0.49 mg/L [IQR = 0.21–1.42] vs 0.30 mg/L [IQR = 0.11–0.66], respectively; *P* = .008) (Figure 1C). The effect of CMV on the CRP concentration was therefore modified by infant HIV exposure status (interaction *P* = .006). For this reason, we modelled the relationship between CMV and CRP separately for HEU and HIV-unexposed infants. On univariable linear regression, CMV DNAemia was not associated with CRP in HIV-unexposed infants (β = −0.75; *P* = .09) but was associated with higher CRP in HEU infants (β = 0.50; *P* = .04).

### Infant Cytomegalovirus and Maternal Human Immunodeficiency Virus Severity

The HEU infants with CMV DNAemia, compared with the HEU infants without CMV DNAemia, had mothers with lower CD4 counts (median = 403 cells/uL [IQR = 277–558] vs 510 [IQR = 354–785], respectively; *P* = .006) and higher HIV loads (median = 9052 copies/mL [IQR = 3186–28642] vs 4222 [IQR = 813–13814]; *P* = .03) (Figure 1D).

### Relationship Between Cytomegalovirus, Inflammation, and Maternal Human Immunodeficiency Virus Severity

Because CMV-positive HEU infants were born to mothers with more advanced HIV than CMV-negative HEU infants, we undertook multivariable linear regression to determine the relationship between CMV DNAemia (as a categorical variable), CRP, and markers of maternal HIV severity (CD4 count and HIV load). The association between CMV acquisition and CRP in HEU infants persisted after adjusting for maternal CD4 count (β = 0.53; *P* = .045), but not after adjusting for log maternal HIV load (β = 0.35; *P* = .27).

## DISCUSSION

This study has 4 key findings. First, prevalence of CMV DNAemia was high at 6 weeks of age in Zimbabwean infants but did not differ by infant HIV exposure status. Second, CMV loads were higher in HEU infants compared with HIV-unexposed infants. Third, HEU infants had a proinflammatory milieu, with CRP concentrations that were >2-fold higher than HIV-unexposed infants. Fourth, we identified a relationship between CMV acquisition, maternal HIV viremia, and inflammation in HEU infants.

Cytomegalovirus acquisition in early life was high in this Zimbabwean birth cohort. Three quarters of infants born to HIV-negative mothers were CMV-positive by 6 weeks of age, similar to previous findings among HIV-infected infants in this cohort [9]. The HEU infants did not have a significantly higher prevalence of CMV than HIV-unexposed infants at 6 weeks. Plasma CMV loads were generally low but were significantly higher in HEU infants compared with HIV-unexposed infants. It is plausible that HEU infants have an impaired ability to control viral pathogens, which may partly explain the increased severity of infections in HEU infants compared with HIV-unexposed infants [1]. Because T-cell abnormalities are one of the most consistently reported findings of immune dysfunction in HEU infants [10], studies comparing CMV-specific immune responses between HEU and HIV-unexposed infants would provide insights into T-cell function in this population.

We found that CRP concentrations were >2-fold higher in HEU infants compared with HIV-unexposed infants, providing further evidence of a proinflammatory state in early life. Previous studies have shown that dendritic cells and monocytes isolated from HEU infants secrete more proinflammatory cytokines compared with HIV-unexposed infants [11] and that CD4 cells from HEU infants have a higher expression of proinflammatory chemokine receptors [12]. Chronic inflammation may be one cause of infection susceptibility in HEU infants, consistent with the link between immune activation and monocyte and T-cell dysfunction in the elderly [13, 14]. In HIV-infected children, high concentrations of inflammatory biomarkers (CRP or interleukin 6) are predictive of mortality [15]. Further studies are needed to dissect the relationship between inflammation and mortality among HEU infants.

The drivers of inflammation and immune activation in HEU infants remain uncertain [2]. Although we have previously reported more morbidity among HEU infants compared with HIV-unexposed infants in the whole ZVITAMBO cohort [2], HEU infants did not have significantly higher prevalence of recent illness in this substudy, except oral thrush, which would be an unlikely cause of elevated CRP. Median CRPs in this study were in the subclinical range, indicating low-grade inflammation. We hypothesised that a higher CMV prevalence among HEU infants might be one cause of inflammation, but the prevalence of CMV DNAemia was similar between groups. However, the relationship between CMV acquisition and inflammation differed between infants with and without HIV exposure. The CMV-positive HEU infants had a higher median CRP than the CMV-negative HEU infants, whereas this relationship was not seen in HIV-unexposed infants. The association between CMV status and CRP persisted after adjusting for maternal CD4 count but disappeared after adjusting for maternal HIV load. These findings suggest that inflammation in HEU infants may be driven in part by exposure to HIV viremia, rather than exposure to an immunosuppressed intrauterine environment per se. Exposure to HIV products across the placenta is consistent with prior studies that have shown evidence of HIV-specific T-cell responses in HEU infants [16]. We hypothesize that exposure to HIV products modulates the developing immune system, either leading directly to infant inflammation or priming the immune system to generate exaggerated inflammatory responses to infection. Whether suppressing maternal HIV load during pregnancy has a positive influence on HEU infant immunology warrants further investigation. However, it is notable that HEU infants appear to have higher morbidity even in the current prevention of mother-to-child transmission of HIV era [1], suggesting there are other drivers of immune dysfunction apart from maternal viremia.

This study has several strengths. First, the HIV status of mothers and infants was well characterized. Second, the participants were recruited before the availability of antiretroviral therapy, making this a “natural history” cohort that could not be replicated. Third, we assessed CMV acquisition using real-time PCR, which has better sensitivity and specificity than serological testing. However, we were unable to differentiate between congenital and postnatal CMV infection, and we looked only in 1 compartment and at 1 point in time, so we may have underestimated the proportion of CMV-infected infants and peak levels of CMV viremia. We may have been underpowered to detect a statistical difference in CMV prevalence between groups, and we could not determine causal relationships between CMV and CRP in this retrospective analysis. Infant survival through 6 months was an inclusion criterion for this substudy, so we could ensure HEU infants were truly HIV uninfected at 6 months; it is possible that excluding infants who died means we missed those with the most severe CMV disease.

In summary, CMV acquisition in early life was high, irrespective of HIV exposure status. The HEU infants had higher CMV loads than HIV-unexposed infants, suggesting a defect in the ability to control the infection. Human immunodeficiency virus exposure was associated with elevated CRP concentrations, building on previous findings of a proinflammatory milieu in HEU infants. Excess inflammation in HEU infants was related to CMV acquisition but appeared to be driven by higher HIV loads in mothers of CMV-positive infants compared with CMV-negative HEU infants. Viral suppression in HIV-infected mothers during pregnancy and breastfeeding may be key to improving outcomes of infants, even when they remain uninfected.

## Notes

***Financial support.*** This work was supported by a grant from Barts Charity (grant no. MGU0300 to C. E.). C. E. is funded by the National Institute for Health Research. A. J. P. is funded by the Wellcome Trust (grant no. 108065/Z/15/Z). The ZVITAMBO trial was supported by the Canadian International Development Agency (CIDA) (R/C Project 690/M3688), United States Agency for International Development (USAID) (cooperative agreement number HRN-A-00-97-00015-00 between Johns Hopkins University and the Office of Health and Nutrition–USAID), and a grant from the Bill and Melinda Gates Foundation, Seattle, Washington. Additional funding was received from the SARA Project, which is operated by the Academy for Educational Development, Washington DC, and is funded by USAID’s Bureau for Africa, Office of Sustainable Development under the terms of contract AOT-C-00-99-00237-00, the Rockefeller Foundation (New York, NY) and BASF (Ludwigshafen, Germany).

***Potential conflicts of interest.*** All authors: No reported conflicts. All authors have submitted the ICMJE Form for Potential Conflicts of Interest. Conflicts that the editors consider relevant to the content of the manuscript have been disclosed.
